# Image-based plant wilting estimation

**DOI:** 10.1186/s13007-023-01026-w

**Published:** 2023-05-31

**Authors:** Changye Yang, Sriram Baireddy, Valérian Méline, Enyu Cai, Denise Caldwell, Anjali S. Iyer-Pascuzzi, Edward J. Delp

**Affiliations:** 1 Video and Image Processing Laboratory (VIPER), School of Electrical and Computer Engineering, Purdue University, 465 Northwestern Avenue, West Lafayette, IN 47907 USA; 2 Department of Botany and Plant Pathology and Center for Plant Biology, Purdue University, 915 W. State Street, West Lafayette, IN 47907 USA

**Keywords:** Machine learning, Image processing, Wilt estimation

## Abstract

**Background:**

Environmental stress due to climate or pathogens is a major threat to modern agriculture. Plant genetic resistance to these stresses is one way to develop more resilient crops, but accurately quantifying plant phenotypic responses can be challenging. Here we develop and test a set of metrics to quantify plant wilting, which can occur in response to abiotic stress such as heat or drought, or in response to biotic stress caused by pathogenic microbes. These metrics can be useful in genomic studies to identify genes and genomic regions underlying plant resistance to a given stress.

**Results:**

We use two datasets: one of tomatoes inoculated with *Ralstonia solanacearum*, a soilborne pathogen that causes bacterial wilt disease, and another of soybeans exposed to water stress. For both tomato and soybean, the metrics predict the visual wilting score provided by human experts. Specific to the tomato dataset, we demonstrate that our metrics can capture the genetic difference of bacterium wilt resistance among resistant and susceptible tomato genotypes. In soybean, we show that our metrics can capture the effect of water stress.

**Conclusion:**

Our proposed RGB image-based wilting metrics can be useful for identifying plant wilting caused by diverse stresses in different plant species.

## Introduction

Plants are continually exposed to abiotic or biotic stress. Phenotypic changes induced by these stresses are indicators of plant health and are informative for plant stress resilience. Accurately quantifying these phenotypic responses to stress enables the identification of genomic regions and genes that function in responding to the stress. However, many plant responses to stress can be challenging to consistently assess with the human eye. One example is plant wilting, which occurs when plants droop in response to heat, loss of water, or disease. Here we develop image-based metrics to quantify plant wilting over time in response to biotic or abiotic stress. We test our metrics in tomato and soybean and show their effectiveness in response to a soilborne bacterial pathogen and water stress.

*Ralstonia solanacearum* (*Rs*) [1] is a soil-borne bacterium that first infects plant roots. Bacteria multiply in the root vasculature and secrete an exopolysaccharide (*EPS*). *EPS* acts like a plug in the xylem, preventing water movement from the root to the shoot. In susceptible plants, this leads to aboveground wilting and eventual death. Resistant plants are colonized by bacteria but at lower levels compared to susceptible plants, and are able to continue growth and development. Bacterial wilt caused by *Rs* [1] is a major threat to crop production worldwide, particularly Solanaceous species such as tomato [2–4]. One of the best methods for controlling bacterial wilt is genetically resistant plants; however, there are few known resistance genes that function against *Rs* [5]. To identify resistance genes, metrics to quantify *Rs*-induced plant wilt are desirable.

Water Stress(WS) has always been a major threat to agriculture, and the problem becomes more challenging with increasing population and climate change [6]. For example, wheat is reported to suffer up to $21$ to $40\%$ yield reductions globally from year 1980 to 2015 [7]. WS affects plant growth, photosynthesis, nutrient and water relations, and often ultimately causes a significant reduction in crop yields [7]. Similar to the studies of identifying Response to *Rs*, metrics to quantify plant wilt can also be useful for studying the effect of WS.

Plant wilting is often visually assessed using a numerical scale (0.0–1.0 for example), where lower numbers indicate fewer percentage of wilted leaves and the highest number indicates total plant wilting. While these scales are useful, it can be challenging to consistently score wilting across populations and among different individuals doing the phenotyping.

In this paper, we propose a set of computational wilting metrics using RGB images of the plant. We tested our metrics on wilt-resistant and wilt-susceptible tomato varieties as well as on soybean plants subjected to water stress. We use machine learning-based methods to show that expert-labeled wilting scores can be predicted using our metrics and that we can predict WS-induced wilting in soybean. Thus, our computer vision-based metrics function across species and against multiple stresses that cause wilting.

There are many ways for plant scientists to quantify the effect of environmental stress [3, 8, 10, 18], but each method has its own shortcomings. One approach is to have experts visually examine the plants to determine wilting. For example, in [8], Engelbrecht et almeasure leaf water potential using visual assessment that is very subjective and difficult to reproduce. For tomato plants, experts rate each plant on its degree of wilting, taking into consideration many plant features such as the overall loss of plant mass and the color shift [12]. In most cases only trained experts can assign a proper visual score. Several other sensor-based wilting metrics in the past have also been proposed. In [14], Caplan et alused manually measured leaf angles as an indicator of WS stress. In [15], Bock et aldetermined disease severity with RGB images of individual leaves. These methods require imaging individual leaves so they are very labor intensive. Other methods such as [16, 17] use RGB images for estimating wilting, but they require special equipment such as guided rail cameras and laser sensors [17] or field servers [16]. Since RGB images are commonly used for many plant studies [13, 16, 17, 19–21], we design our wilting metrics using several RGB images of the plant. Our method will not require any human expert input nor complicated equipment such as guided rail cameras or laser sensors.

Color information has often been used in wilting estimation. For example, in [22], Sancho et alused RGB image-based color information as part of the metrics to estimate *Verticillium* wilt of olive plants. Sancho et alincorporated eighteen color measurements into their metrics, but some of the metrics require cutting the olive leaves. Similar to Sancho et al, the metrics we propose also use RGB image-based color information, but we reduce the number of color-based metrics from eighteen to one and our method does not require cutting physical leaves. We also add a color correction step to account for the difference in imaging conditions. Together, our metrics allow non-destructive and objective measurements of plant wilting.

## Materials and methods

### Plant growth

#### Tomato

*R. solanacearum* resistant *Solanum lycopersicum*, Hawaii7996 (H7996) and *Solanum pimpinellifolium*, accession West Virginia 700 (WV700) as well as the recombinant inbred lines (RILs) derived from crossing the two species were planted in a growth chamber with artificial lighting. Plants were separated into an experimental (Inoculated) group and a control (Mock) group. The experimental (Inoculated) group was inoculated with *Rs* at $10^{8}$ CFU/ml by soil drenching approximately 17 days post germination as described [23]. All plants were imaged on the day before inoculation, and three, four, five, and six days post inoculation (dpi).

**Fig. 1:**
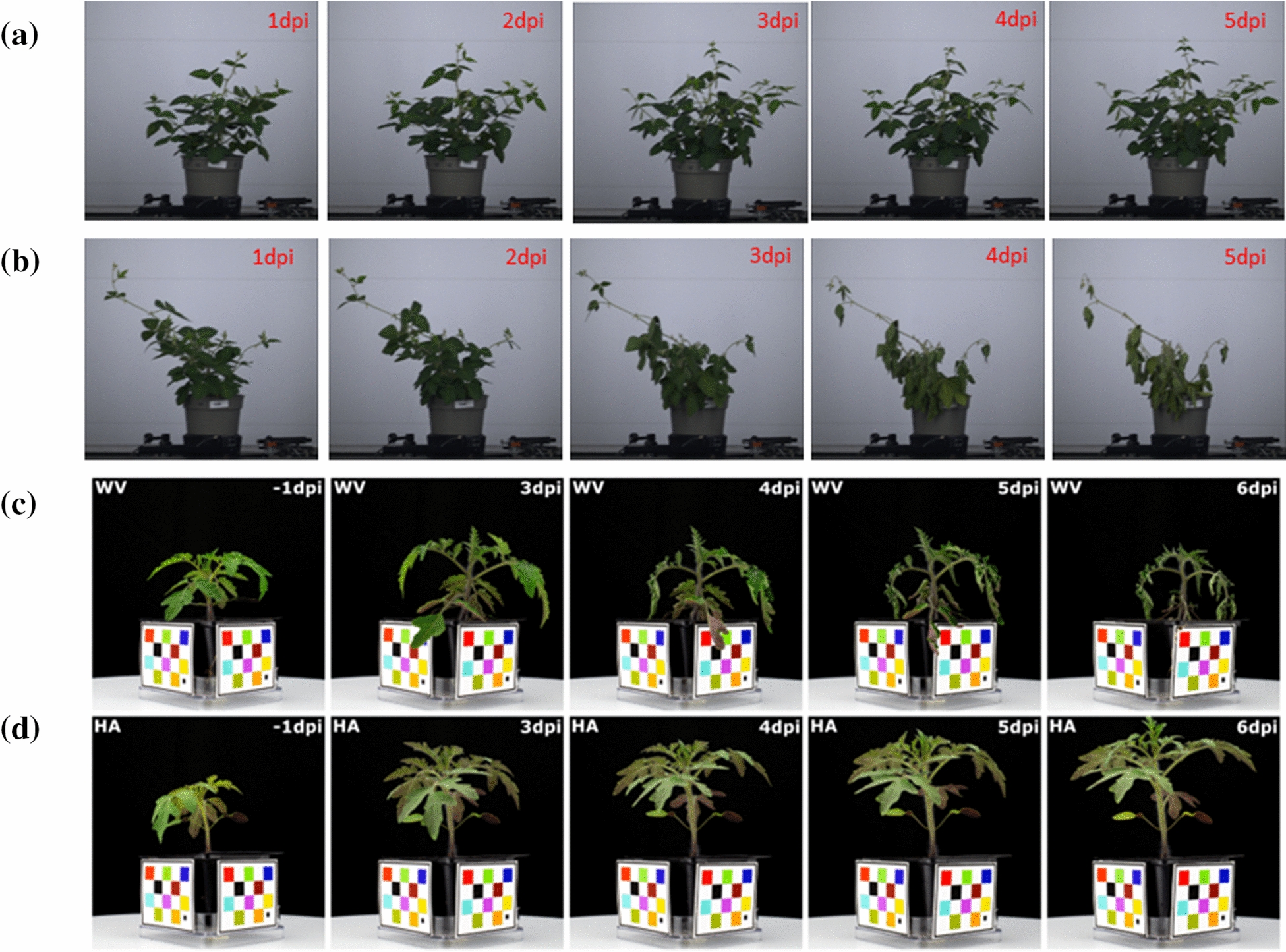
Effect of wilting stress on plants(tomato images are color corrected to show changes in color): **a** Mock soybean plants (*Glycine max KS 5004N*) **b** Soybean plants after stopping irrigation, dpi means days post irrigation **c** *RS* induced wilt on WV700(WV) variant tomato plants, WV700 is the more susceptible variant, dpi means days post inoculation **d** *RS* induced wilt on H7995(HA) variant tomato plants, H7995 is the more resistant variant, dpi means days post inoculation

For all images, we used the same camera positioned at the same location and under controlled lighting conditions. Fiducial markers were used for color correction. Sample images can be seen in Fig. 1. Each time a plant was imaged, eight side-view images were acquired from eight angles. In summary, there were approximately 1000 plants in the inoculated group. Of the 1000 plants, 61 were H7996 and 61 are WV700 and the rest were the offspring species. The mock group contained 36 plants (18 H7996 and 18 WV700). Each image was $5496 \times 3670$ pixels with a spatial resolution of $C_{pres}=0.52$ mm/pixel. Plant experts visually examined the plants eight days after inoculation. They rated each plant relative to its degree of wilting using a continuous score between 0 and 1, where 0 was “no wilting”. The wilting metrics and the associated expert visual wilting scores were split in a 6:4 ratio for training and testing.

#### Soybean

Soybean Plants (*Glycine max KS 5004N*) were grown in a growth chamber in the Ag Alumni Seed Phenotyping Facility (AAPF) at Purdue University. The growth chamber temperature was set to $26^\circ C$, with a relative humidity of $60\%$, with a lighting cycle of 14 hours light per 10 hours dark, PAR: 800 µmol $m^{-2} s^{-2}$. Plants were planted in Berger BM6 All-Purpose media inoculated with *Rhizobium sp.* and grown for 6 weeks before applying water stress by stopping irrigation for 7 days. Sample images can be seen in Fig. 1. 36 soybean plants were imaged, 18 in the mock group and 18 exposed to water stress. The plants were imaged from 12 different angles 1,3, 4, 5, and 6 days post irrigation(dpi). Each image was $2054 \times 2462$ pixels with a spatial resolution of $C_{pres}=0.88$ mm/pixel. We used the same methods as in tomato to extract the wilting metrics from the soybean plants. Due to the absence of the fiducial markers in the soybean dataset, we did not extract the color-based metrics. We used the average of all metrics as the final measurement. We also asked a plant expert to give the plant wilting scores(0, 1, 2, 3) at the end of the experiment.

### Methods overview

Bacterial or WS wilt has a significant impact on the color and shape of plants as seen in Fig. 1. Our primary approach was to estimate color and shape information from RGB images. For color information, we estimated the color distribution of the plant image pixels. For shape analysis, we first used conventional metrics such as width and height. Since the conventional metrics only captured information related to the outer edge of the plants, we developed several stem-based metrics which estimate the distribution of the plant materials relative to the stem.

**Fig. 2:**
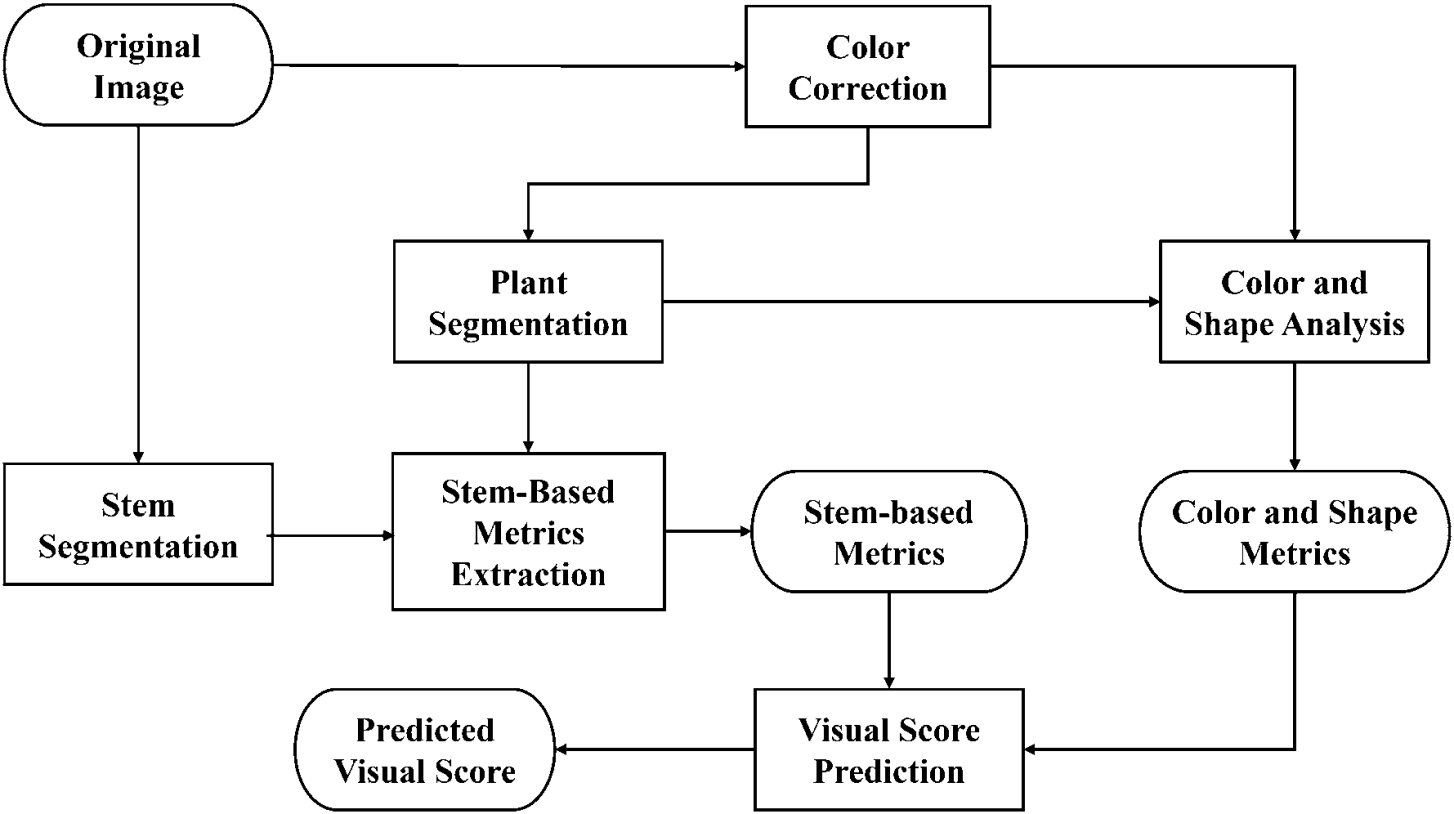
Block diagram of our method

Figure 2 shows a block diagram of our proposed method. Initially, we used color correction, plant segmentation, and stem segmentation on the original RGB images. Color correction was used to address the image color inconsistency caused by the camera settings and acquisition conditions (*e*.*g*., lighting). Plant and stem segmentation were used to capture the plant shape information. These three initial processing steps were required before estimating the wilting metrics.

All metrics could be categorized into color, shape (non-stem) based, and stem-based metrics. Color and shape (non-stem) based metrics had been widely used for plant phenotyping [16, 17]. Here, we describe several additional stem-based metrics to provide more information about plant wilting. Color and shape-based metrics used color-corrected images and the corresponding plant segmentation mask. Stem-based metrics require the plant mask and stem segmentation mask. To demonstrate the utility of our metrics, we also designed a random forest [24] to predict a visual wilting score for a plant using our metrics.

**Fig. 3:**
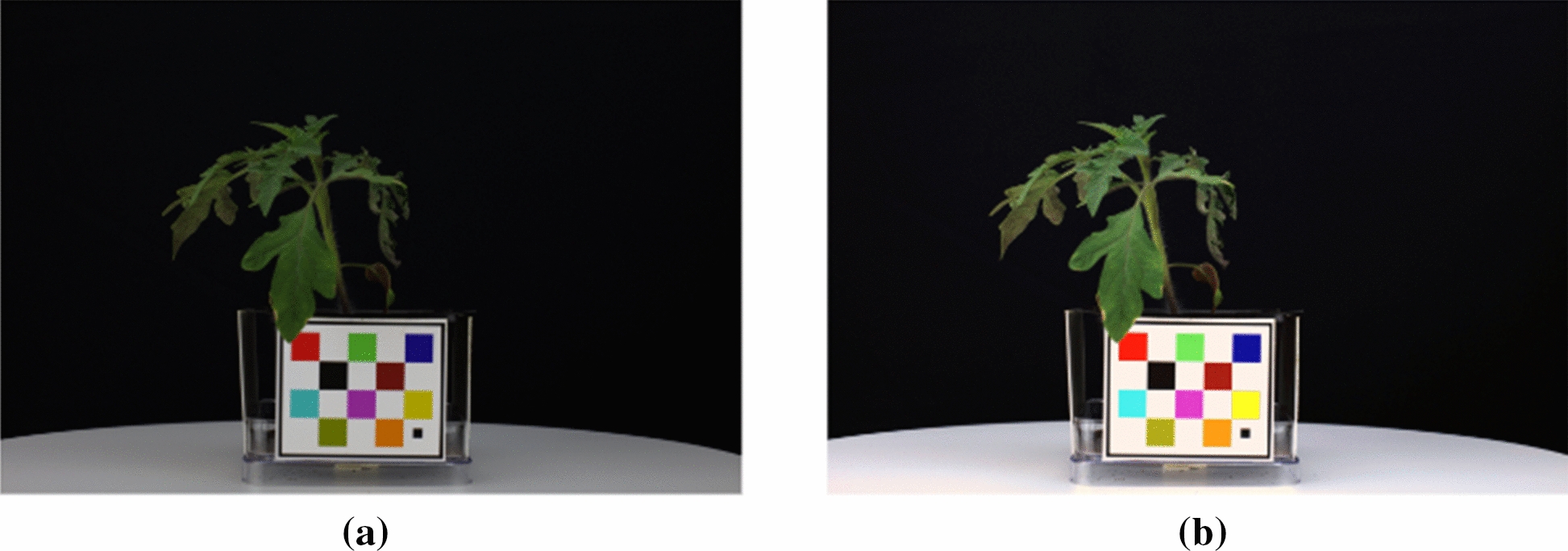
The effect of color correction **a** Original image **b** Color corrected image

### Initial processing

#### Color correction

To suppress the lighting condition variations, images were color corrected using the Fiducial Marker (FM) as a reference object, as seen in Fig. 3). The FM was a colored checkerboard that has known physical dimensions and known colorimetric pixel values for each color square. The FM was detected in the image and the average RGB pixel values for each color square were estimated. Each pixel in the image was subsequently transformed to the correct color using the known actual RGB values of the FM. Let $C_{\text {FM}}$ be a $10\times 3$ matrix which consists of the average R, G, and B pixel values for the 10 colors in the FM. We also knew the 10 colorimetric pixel values of the color squares in the FM, represented by the $10\times 3$ matrix $C_{\text {colorimetric}}$. We estimated the $3\times 3$ color transformation matrix *T* used to correct each pixel value:

1 $$\begin{aligned} C_{\text {colorimetric}}&= C_{\text {FM}} \times T \end{aligned}$$

2 $$\begin{aligned} T&= (C_{\text {FM}}^TC_{\text {FM}})^{-1}C_{\text {FM}}^T \times C_{\text {colorimetric}} \end{aligned}$$

*T* was used to color correct the pixels of the original RGB image. The $N\times 3$ matrix *O* consisted of the R, G, and B pixel values for the *N* pixels in the original RGB image. Then the $N\times 3$ matrix ${\hat{O}}$ was the color corrected pixel matrix, where

3 $$\begin{aligned} {\hat{O}} = O \times T \end{aligned}$$

An example of a color corrected image is shown in Fig. 3.

#### Plant segmentation

Two color channels were selected based on our experiments: (1) the V channel from the HSV color space; and (2) the B* channel from the L*A*B* color space. Plant segmentation masks for these two color channels were obtained by empirically determining separation thresholds for each channel. A threshold of 140 for the V channel and 130 for the B* channel was used, assuming the pixel values are distributed between 0 and 255 in each color channel. The binary segmentation masks were combined using the logical ‘OR’ function to obtain a single segmentation mask. To ensure any undesired objects in the mask are removed, the opening and closing morphological operations [25] with a $3\times 3$ matrix consisting entirely of 1s as the structuring element was used to remove noise and fill holes.

**Fig. 4:**
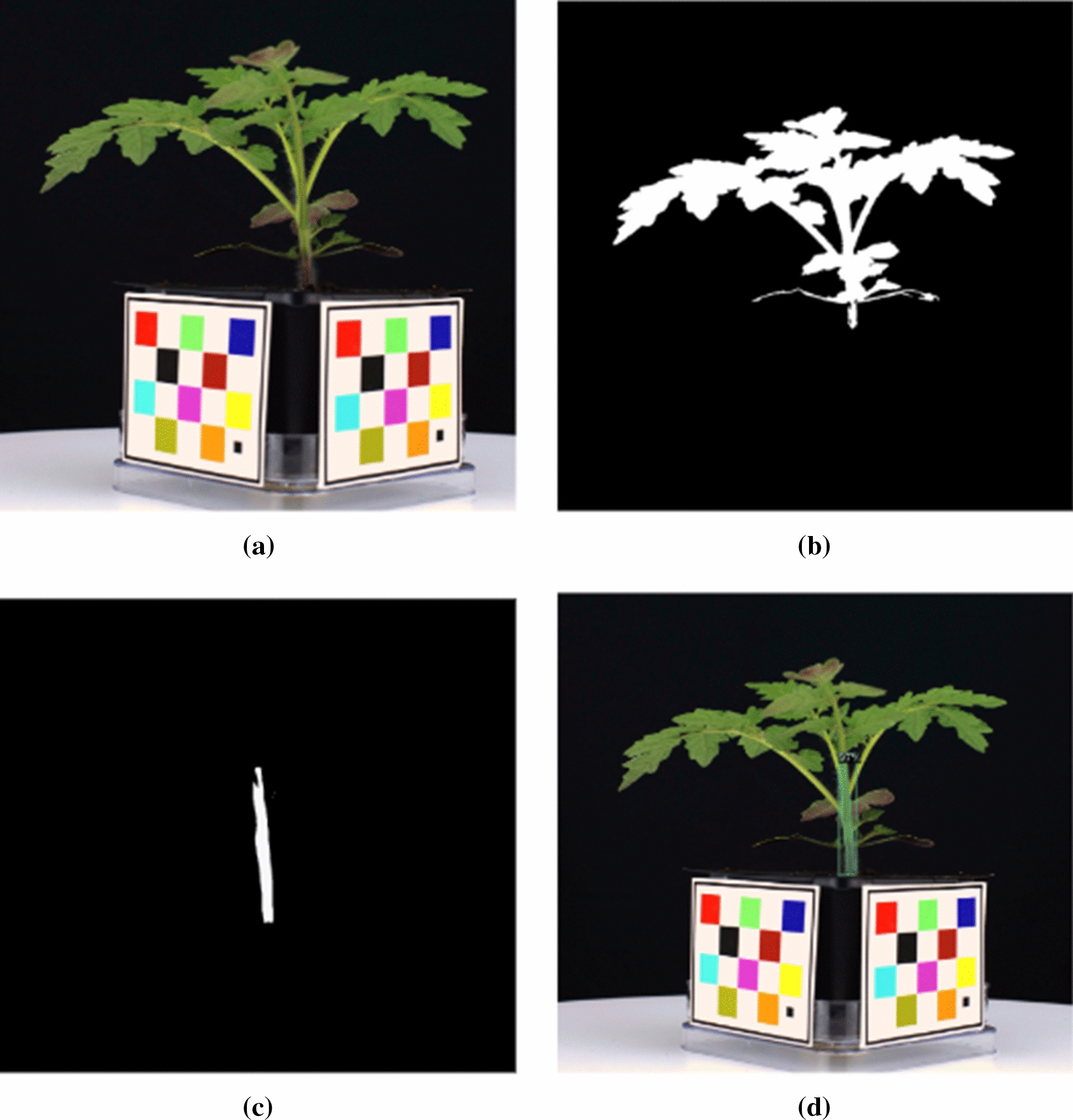
An example of a mock WV700 plant, all images are cropped: **a** Cropped plant image **b** Plant segmentation mask **c** Stem segmentation mask **d** Stem segmentation mask overlaid on plant image

#### Stem segmentation

A deep neural network-based solution similar to what was used by Yang et al. [26] was used for stem segmentation. A set of segmentation stem masks were manually labeled and used to train the stem segmentation networks. Two convolutional neural networks (CNN) [27] were used for stem segmentation, Mask R-CNN [28] and U-Net [29]. An example of the results of the Mask R-CNN network, which was used for most of the stem segmentation, is shown in Fig. 4. For the plants in which Mask R-CNN fails to detect stems, U-Net was used to detect the stem. Mask R-CNN produces better quality [26] masks than U-Net but it sometimes fails to detect the stem.

### Color and shape based metrics

#### Color

After using the plant segmentation mask to capture the pixels containing plant material, the distribution of pixel values in the A* channel of the L*A*B* color space is estimated. Since wilting plants tend to change color from green to brown, the A* channel better captures this plant color variation over time. Differences in the A* pixel distribution were compared over time using the Bhattacharya Distance (BD) [30].

**Fig. 5:**
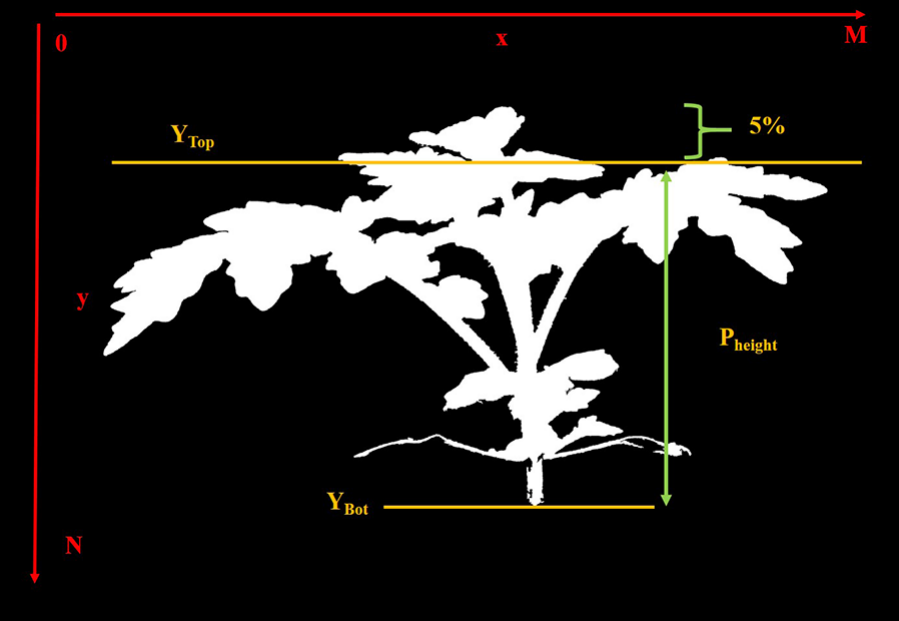
Plant height and index orientation

**Fig. 6:**
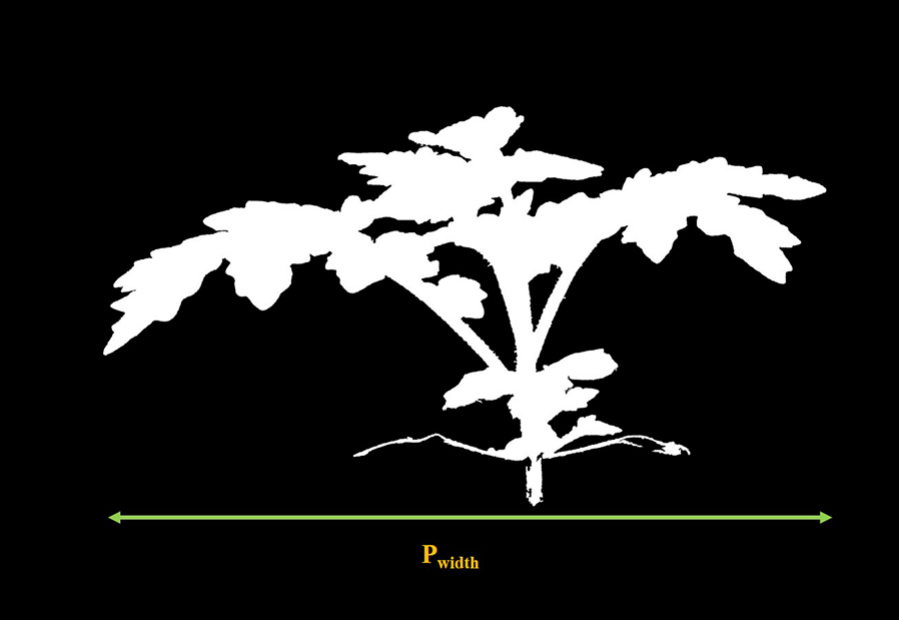
Plant width

#### Plant height, area, and width

Area, the width of the plant segmentation mask, and height were detected from the plant segmentation mask as shown in Fig. 4. Let $p_{msk}(x,y)$ be the plant segmentation mask. The plant area $P_{area}$ (the total area of the plant material) is equal to the number of pixels in $p_{msk}(x,y)$. A horizontal profile $h_{hor}(y)$ at *y* and a vertical profile $h_{ver}(x)$ at x were defined as:

4 $$\begin{aligned} h_{hor}(y) = \sum _{x} p_{msk}(x,y) \end{aligned}$$

5 $$\begin{aligned} h_{ver}(x) = \sum _{y} p_{msk}(x,y) \end{aligned}$$

The plant width $P_{width}$ was defined as the difference between the leftmost pixel and rightmost pixel of the plant mask (indexing orientation shown Fig. 5).

6 $$\begin{aligned} \begin{aligned} P_{width} =\,&P_{max-r}-P_{max-l};\\ where \quad&h_{ver}(P_{max-l}) \ge 1,\\&h_{ver}(P_{max-r}) \ge 1,\\&\sum _{i = 0}^{P_{max-l}-1} h_{ver}(i) = 0,\\&\sum _{i = P_{max-r}+1}^{N} h_{ver}(i) = 0\\ \end{aligned} \end{aligned}$$

For plant height $P_{height}$, the top $5\%$ of plant material was removed. The y-coordinate of the $5\%$ plant material cutoff line as was labeled as $Y_{Top}$, where

7 $$\begin{aligned} \sum _{i=0}^{Y_{Top}} h_{hor}(i) = P_{area} \times 5\% \end{aligned}$$

The upper edge of the pot is defined as the bottom of the plant, denoting its average y-coordinate as $Y_{Bot}$. $P_{height}$ was defined as the difference between the $5\%$ plant material line and the bottom of the plant (Fig. 5).

8 $$\begin{aligned} P_{height} = Y_{Top} - Y_{Bot} \end{aligned}$$

The top $5\%$ of plant material was removed so a small leaf at the top of the plant would not affect the plant height metric.

Figure 5 and Fig. 6 shows an example of $P_{height}$ and $P_{width}$. Plant Height, Area, and Width are general shape metrics of the plant.

### Stem-based metrics

The plant segmentation mask $p_{msk}(x,y)$, and stem segmentation mask $p_{stem}(x,y)$ were used for estimating the distribution. Both the plant segmentation mask and stem segmentation mask are binary images with size $M \times N$ pixels.

**Fig. 7:**
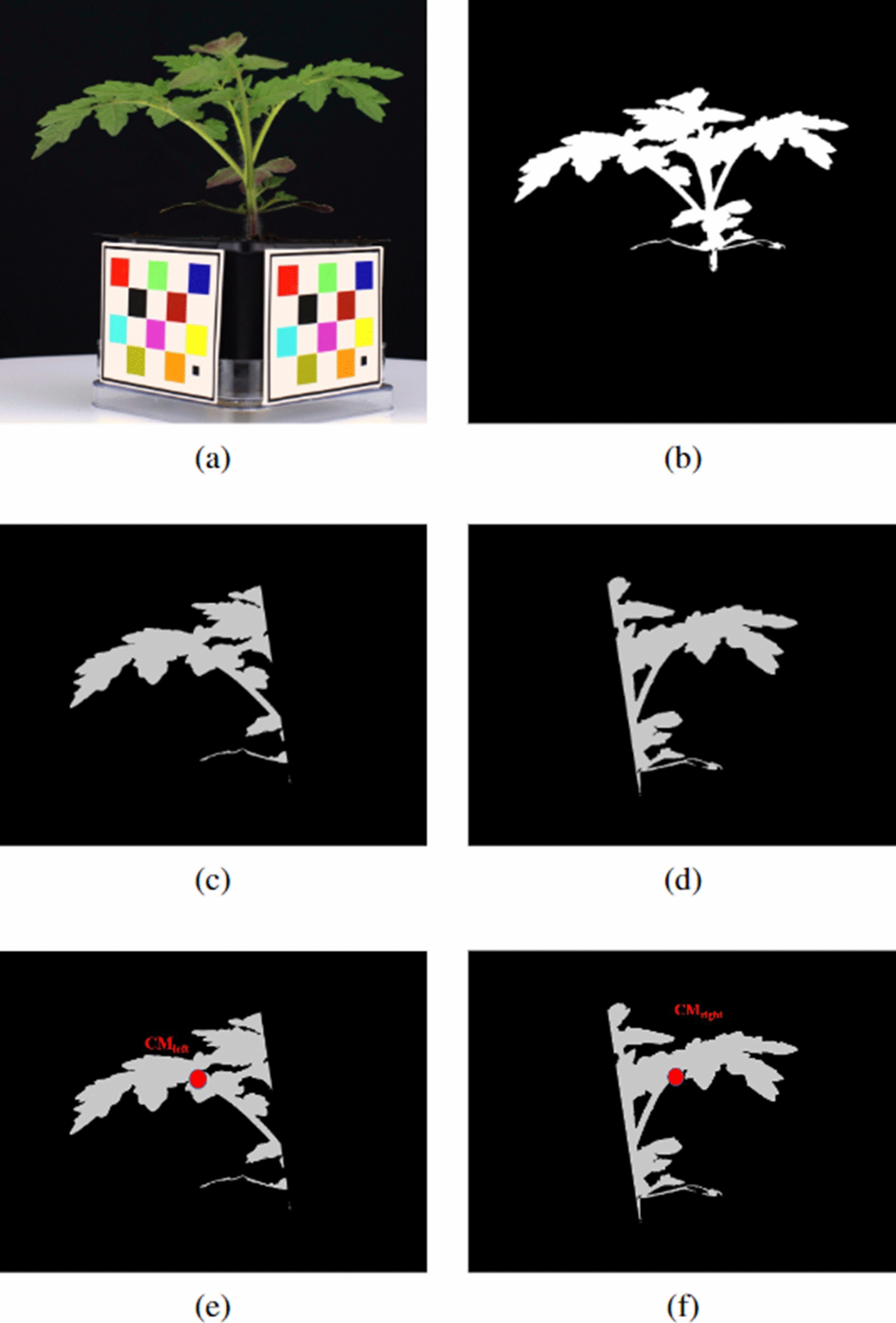
An example of the stem center of mass generation: **a** Original plant **b** Plant segmentation mask **c** Left plant segmentation mask **d** Right stem segmentation mask **e** Left center of mass (CM) **f** Right center of mass (CM)

#### Center of mass

From the stem segmentation mask $p_{stem}$, linear regression [31] was used to form the function $s_{lin}(y)$.

9 $$\begin{aligned} \begin{aligned} s_{lin}(y)&= \alpha + \beta \cdot y \\ \alpha , \beta&= \arg \min _{\alpha , \beta } \sum \limits _{\bar{x},\bar{y}} (\bar{x} - \beta \cdot \bar{y} - \alpha )^{2}\\&\;\qquad \qquad \qquad \cdot p_{stem}(\bar{x},\bar{y}) \\ \end{aligned} \end{aligned}$$

The plant segmentation mask $p_{msk}(x,y)$ was separated using $s_{lin}(y)$ into a left plant mask $p_{l-msk}(x,y)$ and right plant mask $p_{r-msk}(x,y)$ (Fig. 7).

10 $$\begin{aligned} p_{l-msk}(x,y)= {\left\{ \begin{array}{ll} p_{msk}(x,y) &{} \text { if }x \le s_{lin}(y) \\ 0 &{} \text {else} \end{array}\right. } \end{aligned}$$

11 $$\begin{aligned} p_{r-msk}(x,y)= {\left\{ \begin{array}{ll} p_{msk}(x,y) &{} \text {if}\, x > s_{lin}(y) \\ 0 &{} \text {else} \end{array}\right. } \end{aligned}$$

The left center of mass ${CM}_{left}$ and right center of mass ${CM}_{right}$ were then estimated.

12 $$\begin{aligned} {CM}_{left}= & {} \left( \frac{\sum \limits _{x,y}^{}x\cdot p_{l-msk}(x,y)}{\sum \limits _{x,y}^{} p_{l-msk}(x,y)},\frac{\sum \limits _{x,y}^{}y\cdot p_{l-msk}(x,y)}{\sum \limits _{x,y}^{} p_{l-msk}(x,y)} \right) \end{aligned}$$

13 $$\begin{aligned} {CM}_{right}= & {} \left( \frac{\sum \limits _{x,y}^{}x\cdot p_{r-msk}(x,y)}{\sum \limits _{x,y}^{} p_{r-msk}(x,y)},\frac{\sum \limits _{x,y}^{}y\cdot p_{r-msk}(x,y)}{\sum \limits _{x,y}^{} p_{r-msk}(x,y)} \right) \end{aligned}$$

**Fig. 8:**
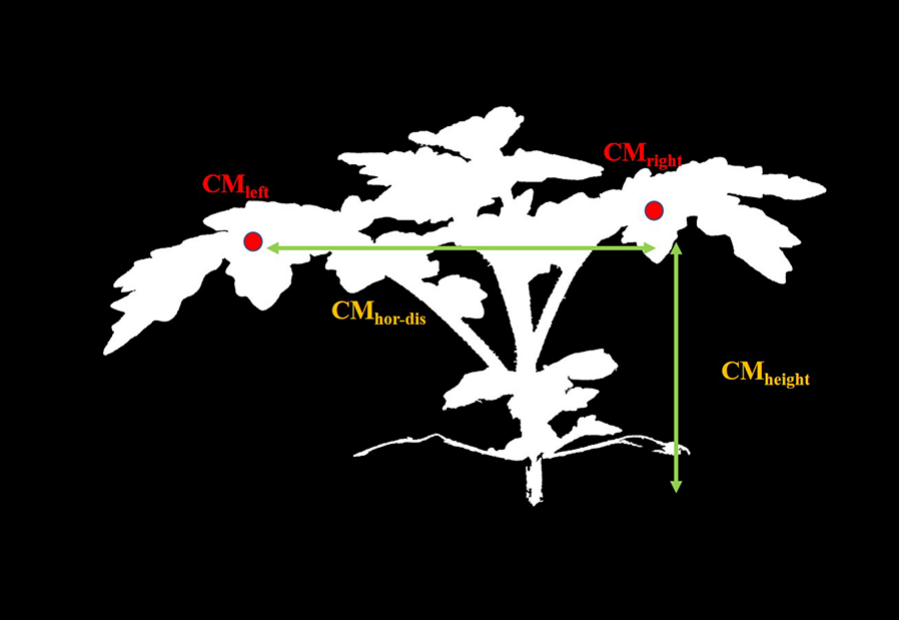
Center of Mass(CM) height and width

The x-coordinate difference between the left and right center of mass (CM) was defined as the Center of Mass Horizontal Distance(CM Width) $CM_{hor-dis}$. The average of the difference between the CM y-coordinates and the bottom of the plant $Y_{Bot}$ is defined as the Center of Mass Height(CM Height) $CM_{height}$. Figure 8 shows a visualization of the metrics.

#### Plant mass vertical and horizontal distribution

Vertical distribution captured the plant material (Mass) distribution along the y-axis for each half of the plant mask and could be sampled at a user-defined percentage. Using $90\%$ as an example, for the left plant mask, the horizontal profile $h_{l-hprof}(y)$ at *y* was estimated from.

14 $$\begin{aligned} h_{l-hprof}(y) = \sum _{x} p_{l-msk}(x,y) \end{aligned}$$

Then find the y-coordinate of the $90\%$ plant mass line $Y_{l-90v}$, where

15 $$\begin{aligned} \sum _{i=0}^{Y_{l-90v}} h_{l-hprof}(i) = \sum _{x,y} p_{l-msk}(x,y) \times 10\% \end{aligned}$$

The same steps were used to find $Y_{r-90v}$ using the right plant masks. The average $90\%$ distribution $V_{90y}$ was defined as

16 $$\begin{aligned} V_{90y} = \frac{Y_{r-90v} + Y_{l-90v}}{2} \end{aligned}$$

**Fig. 9:**
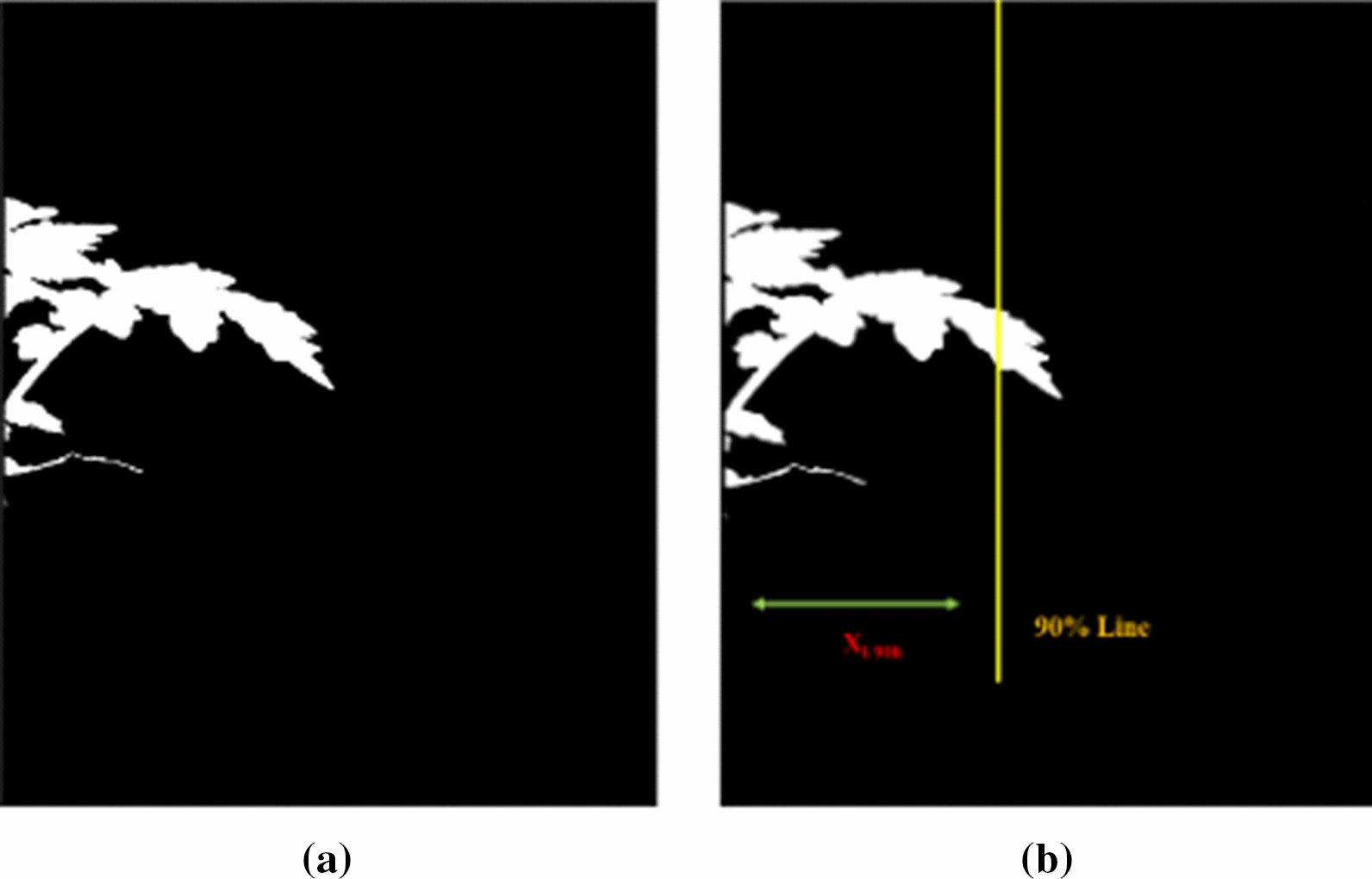
**a** Shifted and flipped left mask $R_{l-msk}(x,y)$ **b** Horizontal $90\%$ plant mass line $X_{l-90h}$

The horizontal distribution captured the plant mass distribution along the x-axis for each half of the plant mask. Because the stem was not always vertical, it was challenging to find an x-coordinate for the stem. First, a horizontal shift to $p_{r-msk}(x,y)$ was performed using the stem separation line, resulting in a shifted $R_{r-msk}(x,y)$. Then $p_{l-msk}(x,y)$ is shifted and flipped resulting in a flipped and shifted $R_{l-msk}(x,y)$ (Fig. 9).

17 $$\begin{aligned} R_{r-msk}(i,y)= & {} p_{r-msk}(slin(y)+i,y) \end{aligned}$$

18 $$\begin{aligned} R_{l-msk}(i,y)= & {} p_{l-msk}(slin(y)-i,y) \end{aligned}$$

The rest of the steps were similar to the vertical distribution calculations discussed earlier. Use $90\%$ as an example, for left half plant masks, the horizontal profile $h_{l-vprof}$ was estimated. The x-coordinate of the $90\%$ plant mass line $X_{l-90h}$ was detected. Similar steps were used to find $X_{r-90h}$ using the right plant masks. The horizontal $90\%$ distribution $H_{90}$ was the sum of $X_{r-90h}$ and $X_{l-90h}$. Figure 9 shows an example shifted mask $R_{l-msk}(x,y)$ and $X_{r-90h}$.

19 $$\begin{aligned} H_{90x} = X_{r-90h} + X_{l-90h} \end{aligned}$$

The shape-based and stem-based metrics described above were then converted from pixel units to metric units using the pixel resolution $C_{pres}$ (millimeters/pixel). $C_{pres}$ was obtained using the known physical dimensions of the Fiducial Marker.

## Results

### Wilting metrics function across species and stresses

In our recent paper [23], we showed that the wilting metrics described above (Plant Area, Plant Height, Plant Width, Center of Mass Horizontal Distance(CM width), Center of Mass Height(CM height), Plant Mass $90\%$ Horizontal Distribution(Xmass), and Plant Mass $90\%$ Vertical Distribution(Ymass)) could differentiate mock and inoculated susceptible tomato plants undergoing Ralstonia-induced wilt (Figure 2 in [23]). In this paper we add another metric based on color, the Bhattacharya Distance (BD) and we show that our metrics function across species and stress.

**Fig. 10:**
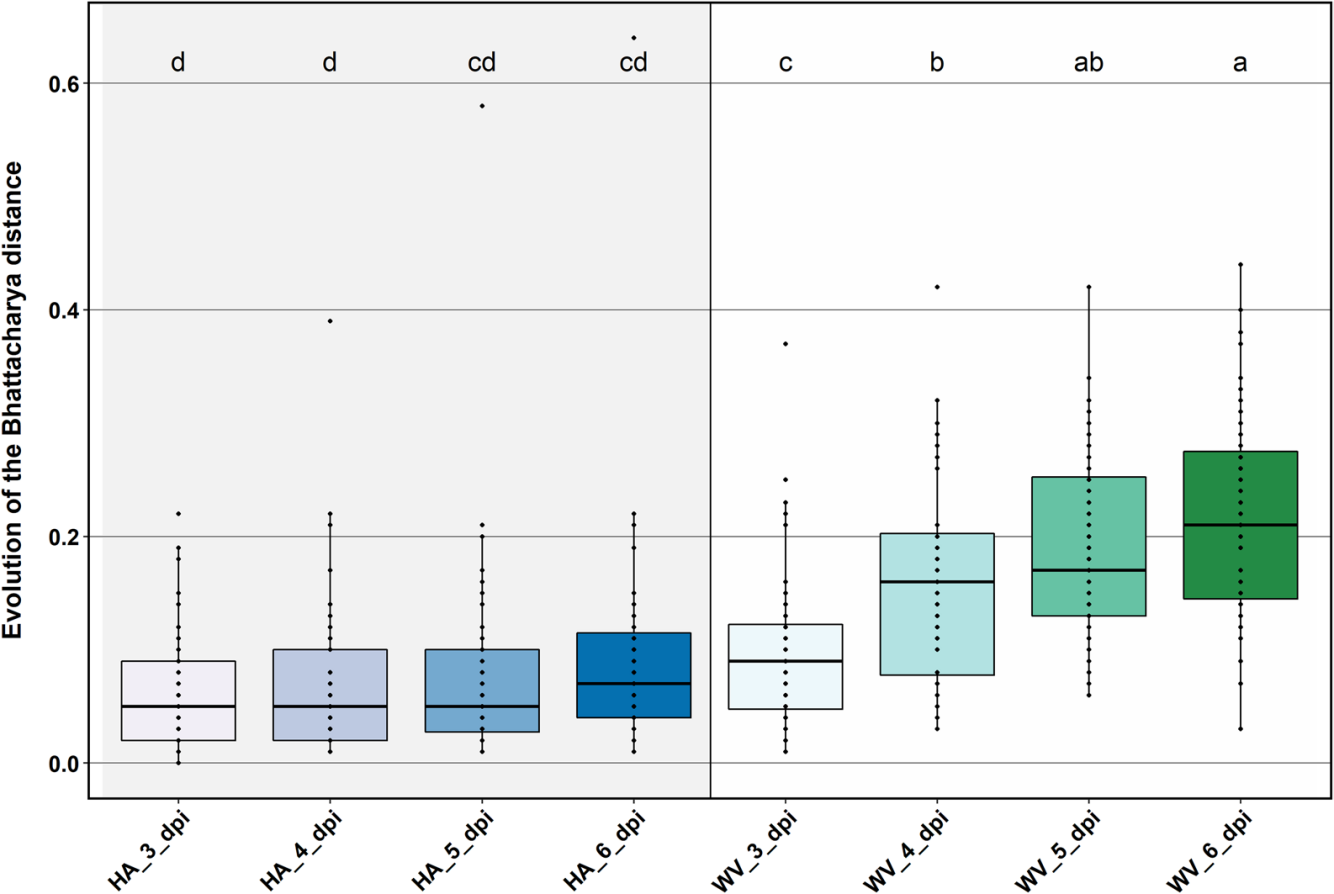
Bhattacharya distance for inoculated H7996 and WV700 plants

Figure 10 shows the Bhattacharya Distance (BD) [30] of the pixel color distributions over time for *RS*-inoculated H7996 and WV700 plants. The BD measures the difference between the pixel color distribution for each day post-inoculation from the pixel color distribution of pre-inoculation plants. The Kruskal-Wallis [32] test for inoculated H7996 plants has a *p*-value of 0.235 and for inoculated WV700 plants the *p*-value is $3.52e-12$. Thus, color does not change in resistant tomato plants but does for susceptible plants.

As shown in Fig. 10, the distribution of the A* pixels in inoculated H7996 plants do not have significant changes in the BD but for inoculated WV700 plants the A* distribution continues to deviate further from pre-inoculation. The results indicate BD is a good indicator of bacterial wilt disease in highly resistant and highly susceptible plants.

**Fig. 11:**
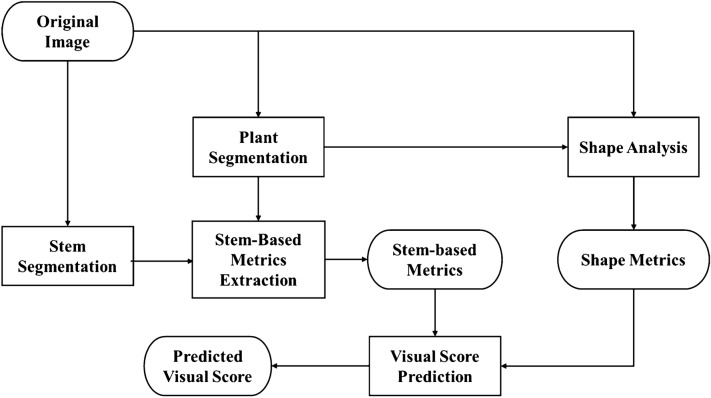
Block diagram of soybean analysis

To demonstrate that our proposed metrics can serve as a general method for quantifying wilt, we experimented with using our metrics to quantify WS-induced wilt on soybean plants. As mentioned in the previous section, due to the absence of the fiducial markers, we did not include any color correction and we also did not extract the color-based metrics. Figure 11 shows the block diagram of the soybean analysis. From visual inspection, the lack of color correction does not affect the quality of plant segmentation since all images are imaged under similar lighting conditions with the same camera. Also based on our experimental results later in this section, we are still able to distinguish between soybean under water stress and mock even without color-based metrics

**Fig. 12:**
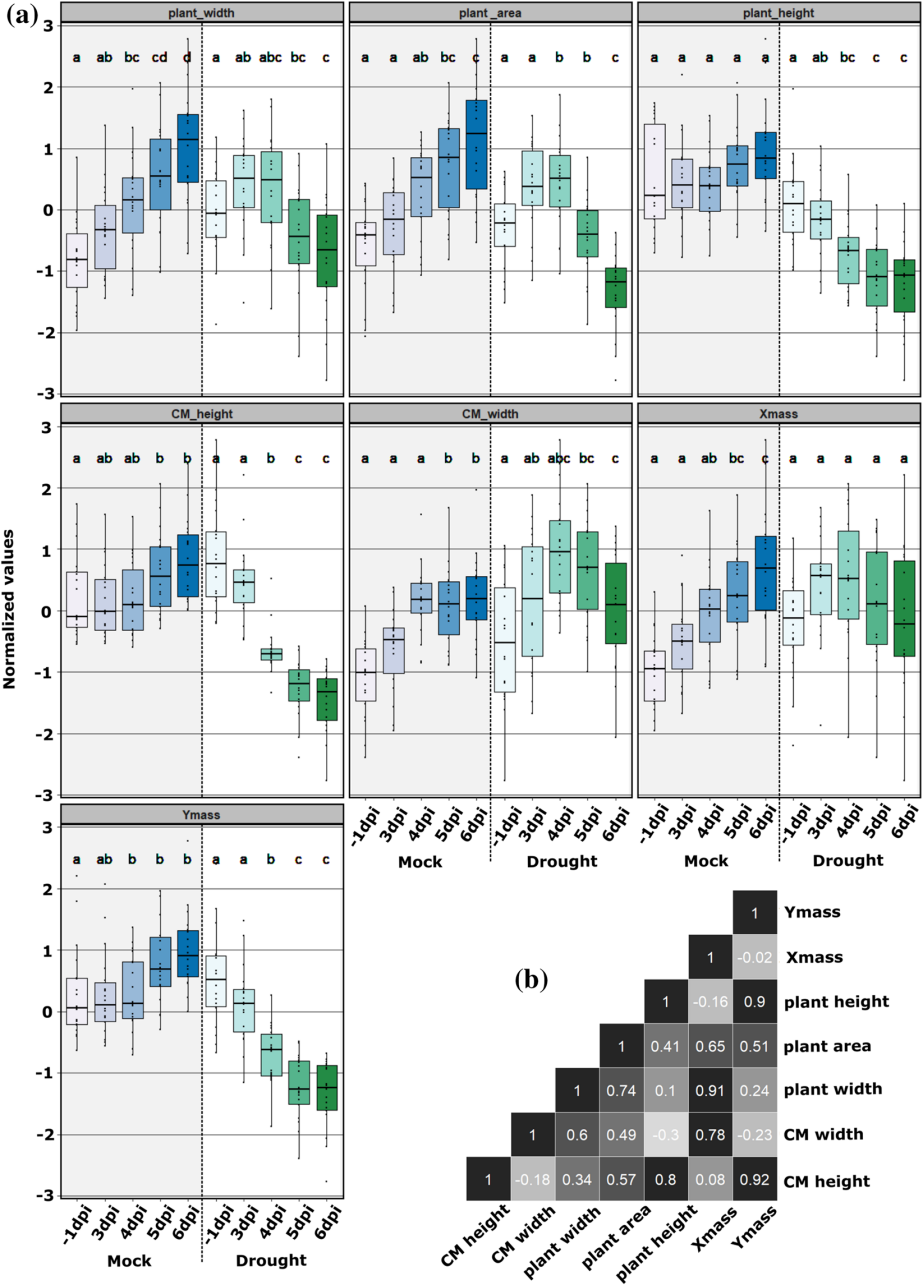
Soybean measurements results (**a**). Plots of each metric over time (**b**). Correlation of each metric

**Table 1:** Soybean metric means(in pixel)

Metrics	1dpi	4dpi	6dpi
CM height mock	332	330	350
CM height WS	350	289	244
CM distance Mock	426	473	475
CM distance WS	450	512	469
Plant area Mock	3.84*e*5	4.36*e*5	4.8*e*5
Plant area WS	4.01*e*5	4.44*e*5	3.35*e*5
Plant height mock	751	752	781
Plant height WS	727	652	612
Plant width mock	599	668	731
Plant width WS	652	679	604
$H_{90}$ Mock	450	494	530
$H_{90}$ WS	485	522	487
$V_{90}$ Mock	611	614	650
$V_{90}$ WS	624	545	498

**Table 2:** Soybean Welch’s t-test results

Metrics	1 vs 4 dpi	1 vs 6 dpi	4 vs 6dpi
CM height mock	0.796	$\mathbf {9.12e-3}$	$\mathbf {3.40e-3}$
CM height WS	$\mathbf {1.17e-9}$	$\mathbf {1.18e-16}$	$\mathbf {1.41e-7}$
CM distance mock	$\mathbf {1.8e-6}$	$\mathbf {4.83e-6}$	0.797
CM distance WS	$\mathbf {7.18e-5}$	0.160	$\mathbf {3.54e-3}$
Plant area mock$^*$	$\mathbf {2.0e-6}^*$	$\mathbf {2.72e-4}^*$	$\mathbf {0.0106}^*$
Plant Area WS$^*$	$\mathbf {3.72e-3}^*$	$\mathbf {4.90e-5}^*$	$\mathbf {4.30e-8}^*$
Plant height mock	0.968	0.081	$\mathbf {0.047}$
Plant height WS	$\mathbf {2.56e-4}$	$\mathbf {8.4e-7}$	$\mathbf {0.048}$
Plant width mock	$\mathbf {8.89e-4}$	$\mathbf {3.05e-7}$	$\mathbf {8.66e-3}$
Plant width WS	0.190	$\mathbf {0.024}$	$\mathbf {2.29e-3}$
$H_{90}$ Mock	$\mathbf {2.29e-4}$	$\mathbf {5.18e-7}$	$\mathbf {0.013}$
$H_{90}$ WS	$\mathbf {0.022}$	0.895	0.065
$V_{90}$ Mock	0.867	$\mathbf {1.94e-3}$	$\mathbf {1.52e-3}$
$V_{90}$ WS	$\mathbf {7.91e-7}$	$\mathbf {4.39e-11}$	$\mathbf {6.05e-4}$

The values are bolded because the results are under 5% significance level so we can reject the null hypothesis

$*$For Plant Area$^*$, we use Rank-Sum test since the 6dpi data failed the normality test

We first investigated whether our metrics could detect WS-induced wilting (Fig. 12). We observed significant differences between mock plants and WS plants for each metric. Figure 12 shows the plots of our metrics on mock and WS-affected soybean plants. Table 1 shows the mean of our metrics and Table 2 shows Welch’s t-test [32] results for soybean plants. From the metric means and the results of the statistical tests, we could observe the trend of each metric under mock and water stress.

CM Height, Plant Height, Plant Width, and Ymass increased over time for the mock group but decreased over time for WS group. CM Width and Plant Area increased over time for the mock group, but the metrics first increased and then decreased for the WS group. We could observe that all metrics increase over time for mock plants and most metrics (all but Xmass) eventually decrease over time for WS plants. The Xmass increased over time for mock, and for WS group it initially increased but later stopped increasing. When the plants were growing in mock condition, we would expect all metrics to increase due to the increasing size. When the plants were under water stress, we would expect all metrics to eventually decrease due to the shrinking size. The behavior of the metrics was consistent with our expectations, we could then conclude that our metrics could capture WS-induced wilting on soybean plants.

When inspecting the metrics more closely, for both tomato and soybean plants, width-dependent metrics such as Plant Width, CM Width, and Xmass are closely correlated to each other, also height dependent metrics such as Plant Height, CM height, and Ymass are closely correlated to each other. Width-dependent metrics such as Plant Width, CM Width, and Xmass demonstrated more changes once under wilting stresses for tomato plants(Figure 3 in [23]). But for soybean plants, height-dependent metrics such as Plant Height, CM height, and Ymass demonstrated more changes once under wilting stresses, as shown in Fig. 12.

### Random forest trained with wilting metrics can predict expert rating of plant stress in tomato and soybean

We investigate whether visual scores assigned by plant experts could be derived from our metrics. A random forest (RF) [24] is used to predict expert visual wilting scores from the wilting metrics. The wilting metrics and the associated expert visual wilting scores are split in a 6:4 ratio for training and testing. Because the tomato expert visual score is given as a continuous value between 0 to 1 and the soybean expert visual score is given as 0, 1, 2, and 3, for both tomato and soybean plants, we perform both regression and classification using the visual score data. For regression, we use visual score data as ground truth and trained the model. For classification, we divide the plants into different classes based on their visual score.

Starting with tomato plants, we examine two scenarios for classification: the visual scores divided into (1) two classes (visual score $0 - 0.5$ as class I, $0.5 - 1.00$ class II); and (2) three classes (visual score $0 - 0.33$ class I, $0.33 - 0.66$ class II, $0.66 - 1.00$ class III). We then train the network for both the two and three classes scenarios. The two classes scenario resulted in a more balanced data split while the three classes scenario avoided the problem of being a binary classification.

The predicted visual scores from all networks are compared with the visual scores assigned by expert plant scientists. For the regression network, we use Mean Absolute Error (MAE) and Mean Squared Error (MSE) as measurements of performance. The accuracy of the classification networks is evaluated using F1 Score [33]:

20 $$\begin{aligned} \text {Precision}=\, & {} \frac{\text {TP}}{\text {TP}+\text {FP}}, \end{aligned}$$

21 $$\begin{aligned} \text {Recall}= \,& {} \frac{\text {TP}}{\text {TP}+\text {FN}}. \end{aligned}$$

22 $$\begin{aligned} \text {F1 Score}= \,& {} \frac{2 \times \text {Precision}\times \text {Recall}}{\text {Precision}+\text {Recall}} \end{aligned}$$

The definition of True Positive(TP),True Negative(TN), False Positive(FP), False Negative(FN), MAE, and MSE can be found in [33]

**Table 3:** Tomato plants Random Forest(RF) classification and regression results

Training type	F1	MAE	MSE
Two classes RF	0.84	N/A	N/A
Three classes RF	0.72	N/A	N/A
Random forest regression	N/A	0.195	0.071

**Fig. 13:**
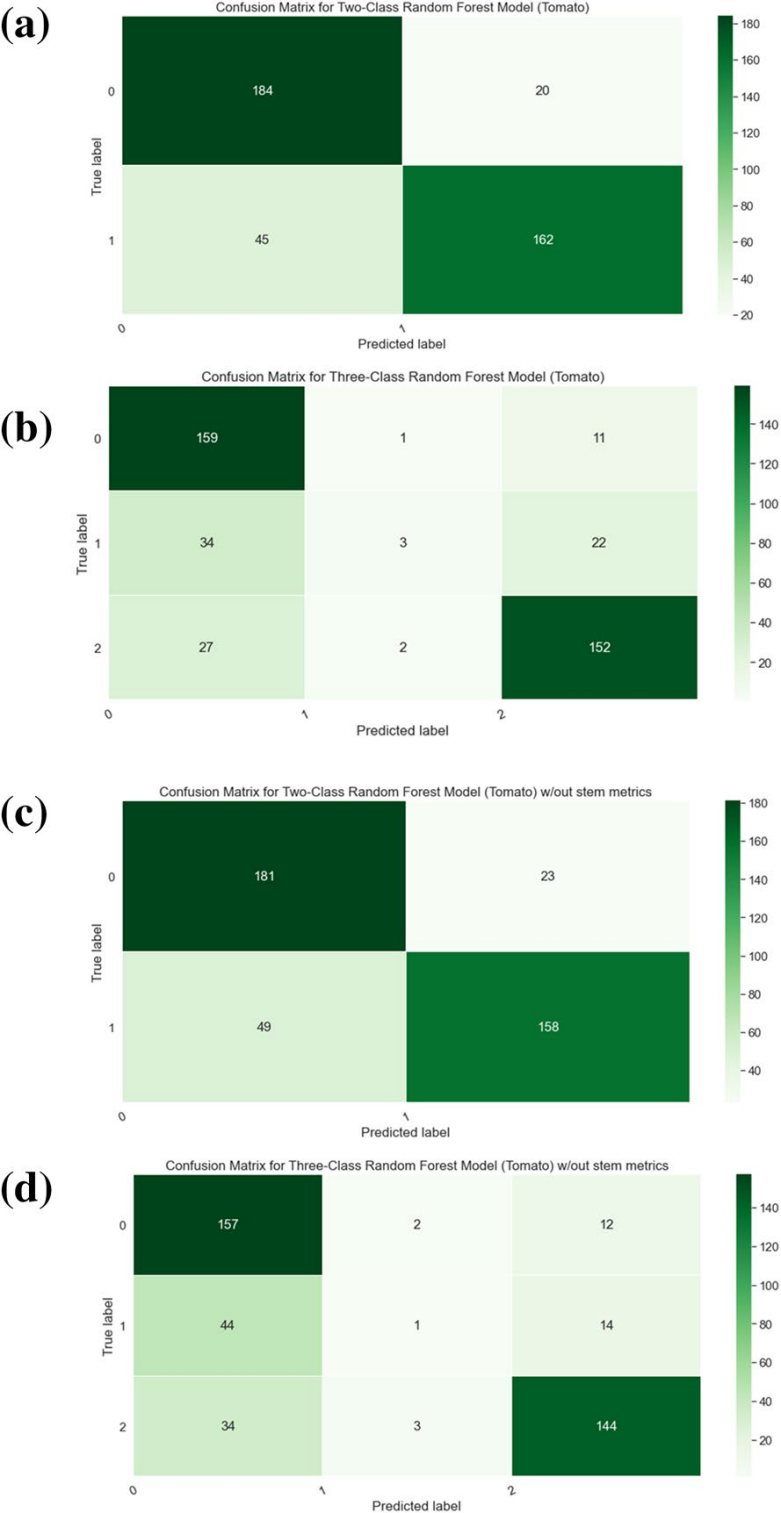
Confusion matrices for tomato plants, we record the total number of plants that fall under each case (TP, TN, FP, FN). **a** Confusion matrix for Two-Class Random Forest (**b**). Confusion matrix for Three-Class Random Forest (**c**). Confusion matrix for Two-Class Random Forest without stem metrics (**d**). Confusion matrix for Three-Class Random Forest without stem metrics

The results of the classification are shown in Table 3. For the two class scenario, the random forest achieved the F1 score of 0.84 and for the three classes scenario the random forest achieved the F1 score of 0.72. The confusion matrices capturing the per-class results of the random forest models are provided in Fig. 13. The drop in performance when moving from the two class to the three class scenario can be attributed to the class imbalance present with the creation of the third class, making it more likely for its samples to be misclassified. The results of the regression are shown in Table 3. The results show that we can capture the expert labeled visual score with an MAE of 0.195 and MSE of 0.071.

The visual scores determined by the random forest are a good predictor of the visual scores provided by the plant experts for every plant. Note that the plant expert visual scores are based on the state of the plant at eight days post-inoculation. Our networks are able to generate visual metrics from images up to six days post-inoculation, meaning that the random forest is able to predict the state of the plant two days in advance.

**Table 4:** Tomato plants Random Forest(RF) classification metrics rank

Metrics	Two classes RF	Three dlasses RF	Regression
Xmass	2	1	1
Plant width	1	2	2
CM width	3	3	3
BD	6	6	6
CM height	7	4	4
Plant areas	4	5	5
Plant height	7	7	7
Ymass	8	8	8

For random forests, we can also find how much each metric contributes to the final prediction. We have attached the rank of the contribution of each metric in Table 4.

**Table 5:** Tomato plants Random Forest (RF) stem and non-stem based metrics comparison results

Training type	F1	MAE	MSE
Two classes RF	0.84	N/A	N/A
Two C RF w/out stem	0.82	N/A	N/A
Three classes RF	0.72	N/A	N/A
Three classes RF w/out stem	0.69	N/A	N/A
Random forest regression	N/A	0.195	0.071
Random forest regression w/out stem	N/A	0.208	0.079

There are concerns that our stem-based metrics are capturing the same information as the shape-based metrics thus the stem-based metrics are not needed. To examine this claim, we also train the classification network without the stem-based metrics, the performance of which is reported in Table 5. We provide the confusion matrices for the random forests trained without the stem metrics in Fig. 13. Similarly we also train the regression network without the stem-based metrics and report the results in Table 5. These results show that stem-based measurements improve the prediction accuracy for both classification and regression networks.

**Table 6:** Regression results from different networks

Training type	MAE	MSE
Random forest(RF)	0.195	0.071
SVM	0.203	0.081
VGG	0.311	0.095

In addition, we compare the performance of random forest networks with SVM [34] and VGG [27] networks in the regression scenario. We choose to use regression because the tomato expert visual score is given in a continuous value between 0 to 1. The input of the SVM is our generated wilting metrics and the input of the VGG is the original images. The results are shown in Table 6. The random forest performs the best among all three networks.

**Table 7:** Classification and regression results for random forest(RF)

Training type	F1 score	MAE	MSE
RF two classes	0.90	N/A	N/A
RF multi classes	0.83	N/A	N/A
RF regression	N/A	0.055	0.008

**Fig. 14:**
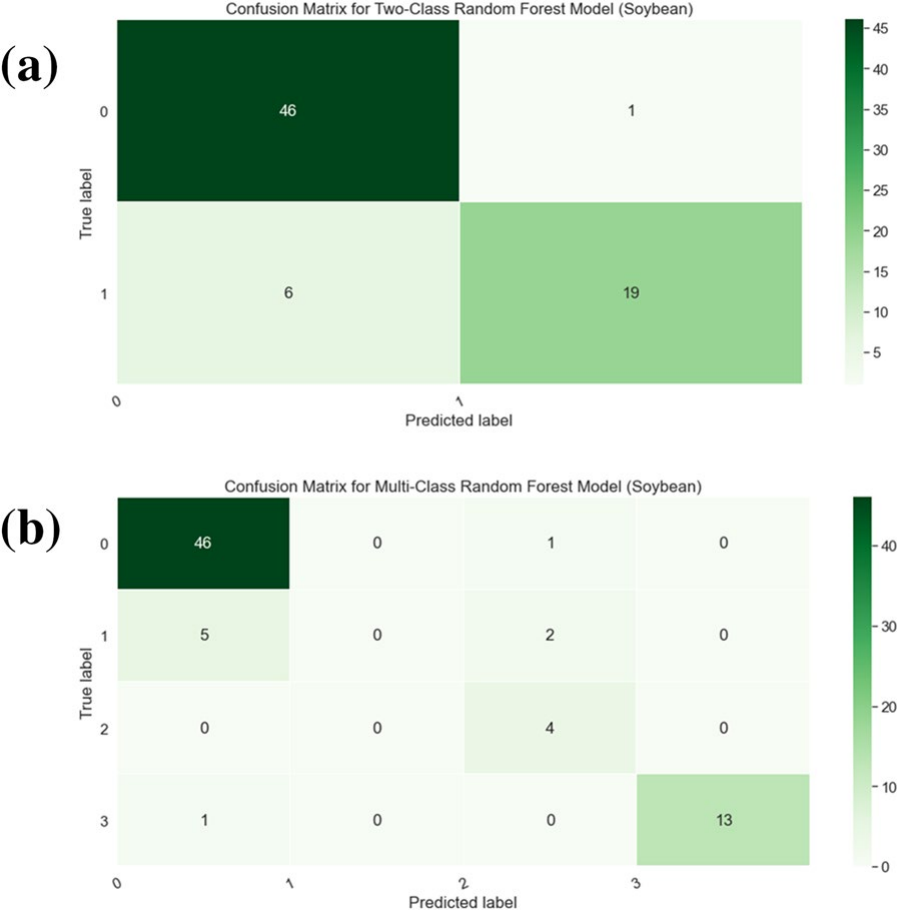
Confusion matrices for soybean plants, we record the total number of plants that fall under each case (TP, TN, FP, FN). **a** Confusion matrix for Two-Class Random Forest (**b**). Confusion matrix for Multi-Class Random Forest

To test whether the random forest network was equally effective in soybean, we trained a random forest(RF) [24] to predict the expert visual wilting scores from our wilting metrics. The results are shown in Table 7. The wilting scores are given in 0, 1, 2, and 3 where $0 =$ no wilt and $3 =$ max wilt. Results were classified in two ways, First, the wilting score 1,2,3 was combined into one wilting class and plants with score of 0 were in the non-wilt class. This two-class classification method resulted in a more balanced dataset between the wilt and non-wilt classes. The random forest of the two-class method has a $90\%$ F1 score for predicting the expert classification results. The second classification method used the original wilting score with four categories. This multi-class method resulted in an $83\%$ F1 score. The confusion matrices capturing the per-class performance of the random forests are provided in Fig. 14. The drop in performance upon moving from the two class scenario to the multi class scenario can be attributed to a class imbalance, leading to more misclassifications for the lesser represented classes. For regression, we assigned a wilting value between 0 to 1 based on the expert wilting score. The random forest gives an MAE [33] of 0.055 and MSE [33] of 0.0077. Thus we can conclude that similar to the tomato dataset, our metrics could predict the expert wilting score for WS-induced wilting on the soybean plants. The rank of metrics contribution is attached in Table 8.

**Table 8:** Soybean plants random forest(RF) classification metrics rank

Metrics	Two classes RF	Multi classes RF	Regression
Xmass	6	6	6
Plant width	7	7	7
CM width	3	4	3
CM height	1	1	1
Plant areas	5	3	5
Plant height	4	5	4
Ymass	2	2	2

Once we inspect the random forest rank of metrics more closely, for tomato plants the width-based metrics such as plant width or CM width are ranked higher than height-based metrics such as plant height or CM height. But for soybean plants, the height-based metrics are ranked higher than width-based metrics. This result is consistent with our findings in the previous section when we evaluate our metrics and the statistical test results directly. We observed that once under wilting stresses, width-based metrics are affected more for tomato plants and height-based metrics are affected more for soybean plants. This could be due to the differences in shoot architecture between tomato and soybean.

## Discussion and conclusion

Here we proposed eight image-based wilting metrics for estimating wilting in plants exposed to stress. The wilting metrics described here can be used to detect wilting in different species from different types of external stresses. They can differentiate wilted from non-wilted plants and thus resistant from susceptible plants. Instead of an arbitrary score estimated by the expert, the wilting metrics described here could provide direct information relating to the physical traits of the plant such as color, shape, or plant mass distribution. The additional physical information could lead to observations that otherwise could not be concluded from a simple expert visual score. For example, if the Bhattacharya Distance of a group of plants with a specific gene increases after inoculation, one could say that this gene might lead to less resistance to Rs induced wilting. In addition, one or more metrics could be used to conduct more comprehensive studies such as QTL analysis as in [23].

Because our pipeline requires clearly defined stems, we speculate that it will work better for crops with clearly defined rigid stems such as tomatoes. For bush-like crops such as blueberry, an improved stem detection method might be needed. Also, since our method only requires RGB images as input, it has the potential for field-based implementation using mobile devices. However, there might be some challenges. Our metrics depend on the segmentation of the plants, so it might not perform well on field crops that are planted close to each other with overlapping plant material. An improved plant segmentation method will be needed. Our current method also requires plant images from multiple angles. For field applications, the number of images required per plant needs to be reduced. In addition, the imaging angle may vary between images, so some image rectification might be needed.

For future work, study could be done on the expert bias of our visual score. We could also evaluate whether our method would be able to differentiate wilting caused by pathogen or water stress. This could potentially be achieved using hyperspectral images. In addition, we could investigate new wilting metrics that can capture true 3D information and could be used for field-based plants. To conclude, we proved the effectiveness of our proposed metrics in quantifying wilt from different causes (bacterium or WS) and on different plant species (soybean and tomato). Also compared to the traditionally expert-labeled wilting scores, our metrics are based on plant physics and are less prone to subjective changes.

## Data Availability

Dataset and software will be made available upon request. Address all correspondence to Edward J. Delp, ace@ecn.purdue.edu
